# Postoperative headache after surgical treatment of cerebellopontine angle tumors: a systematic review

**DOI:** 10.1007/s00405-021-06627-6

**Published:** 2021-02-01

**Authors:** Louis Pogoda, Jelle S. Nijdam, Diederik P. J. Smeeing, Eduard H. J. Voormolen, Fuat Ziylan, Hans G. X. M. Thomeer

**Affiliations:** 1 Department of Otorhinolaryngology-Head and Neck Surgery, University Medical Center Utrecht, Utrecht University, Heidelberglaan 100, 3584 CX Utrecht, The Netherlands; 2 Department of Surgery, University Medical Center Utrecht, Utrecht University, Utrecht, The Netherlands; 3 Department of Neurosurgery, University Medical Center Utrecht, Utrecht University, Utrecht, The Netherlands; 4 Department of Otorhinolaryngology-Head and Neck Surgery, Amsterdam Public Health Research Institute, Amsterdam University Medical Center, Vrije Universiteit Amsterdam, Amsterdam, The Netherlands; 5 Brain Center Rudolf Magnus, University Medical Center Utrecht, Utrecht University, Utrecht, The Netherlands

**Keywords:** Cerebellopontine angle tumors, Vestibular schwannoma, Postoperative headache, Translabyrinthine approach, Retrosigmoid approach, Surgical techniques

## Abstract

**Purpose:**

Postoperative headache (POH) is a complication that occurs after surgical resection of cerebellopontine angle (CPA) tumors. The two most common surgical approaches are the translabyrinthine (TL), and retrosigmoid (RS) approach. The objective of this systematic review was to investigate whether POH occurs more frequently after RS compared to TL approaches.

**Methods:**

A systematic search was conducted in Cochrane, Pubmed and Embase. Studies were included if POH after CPA tumor removal was reported and both surgical approaches were compared. The methodological quality of the studies was assessed using the Risk Of Bias In Non-randomized Studies of Interventions (ROBINS-I) tool.

**Results:**

In total, 3,942 unique articles were screened by title and abstract. After the initial screening process 63 articles were screened for relevance to the inquiry, of which seven studies were included. Three studies found no significant difference between both surgical approaches (*p* = 0.871, *p* = 0.120, *p* = 0.592). Three other studies found a lower rate of POH in the TL group compared to the RS group (*p* = 0.019, *p* < 0.001, *p* < 0.001). Another study showed a significantly lower POH rate in the TL group after one and six months (*p* = 0.006), but not after 1 year (*p* = 0.6).

**Conclusion:**

The results of this systematic review show some evidence of a lower rate of POH in favor of the TL approach versus the RS approach for CPA tumor resection. Prospective research studies are needed to further investigate this finding.

## Introduction

Cerebellopontine angle (CPA) tumors account for around 10% of all intracranial neoplasms [1]. In approximately 98% of the cases, tumors of the CPA are either vestibular schwannomas (80 to 95%) or meningiomas (5 to 15%) [2, 3]. Despite their non-malignant nature, these tumors might induce severe comorbidity and a range of symptoms (i.e. instability and vestibular problems, cranial nerve neuropathy, intracranial hyperpressure, sensorineural hearing loss, tinnitus and headache). Treatment options of CPA tumors encompass observation (wait and scan), radiotherapy or a surgical approach.

The operative management of CPA tumors consists mainly of two different surgical approaches: the translabyrinthine (TL), and retrosigmoid (RS) approach [2, 4]. Both of these techniques are employed for any size of tumor, though only the RS approach can be used in cases of hearing preservation surgery. In the minority of cases a third technique, the middle cranial fossa (MF) approach, might be employed in cases of tumors limited to the internal auditory canal (IAC) (or with a minimal CPA extension), with serviceable hearing [5–9]. A disadvantage of the RS approach is that cerebellar retraction is often required to provide enough surgical exposure. Disadvantage of the TL approach is that it leads to total deafness. Despite the advantages and disadvantages of each approach, the main goal of surgical treatment, a (near-total) resection of the tumor with maximum preservation of facial nerve function, could be strived for with all three methods [2, 4, 9, 10].

Postoperative headache (POH) is a known adverse event after CPA surgery, with a significant impact on quality of life [11]. Besides the burden for the individual, chronic pain has a great economic impact [12, 13]. In approximately 65% of patients with POH, it lasts beyond the initial postoperative period [4]. Although 84% of POHs resolve within the first 12 months after surgery, approximately 16% of patients still present with invalidating (refractory) pain even one year after surgery [14]. The reported rates of POH vary greatly between the different approaches (TL: 0 to 84%, RS: 10 to 93%), which might be due to heterogeneity between study populations and differences in POH definitions and study methodology [9, 14–21]. Two systematic reviews identified a trend of less POHs within the group of patients who underwent the TL approach [9, 21]. However, the results of these reviews were mainly based on single arm studies which were highly heterogeneous and difficult to compare. The goal of this review was to investigate the difference in POH rates after the two most applied surgical techniques (TL versus RS) of CPA tumor resection.

## Materials and methods

The review was performed in accordance with the Preferred Reporting Items for Systematic Reviews and Meta-Analyses (PRISMA) statement [22, 23].

### Search strategy

In this systematic review an electronic search was performed using the Cochrane, PubMed and EMBASE databases on 19/10/2020. Keywords used for the search included various synonyms and types for the surgical approach and for CPA tumors. The search strings can be found in the Appendix table.

### Selection criteria

Titles and abstracts were screened independently by two authors. After title and abstract screening, potentially valuable articles were read in full text. Articles were included if written in English, Dutch, German, French, Spanish or Turkish language. Comparative studies were included if postoperative headache after CPA tumor removal was reported for both TL and RS approach. Studies were excluded if only one surgical approach was studied, if it concerned animal studies, opinion papers, poster presentations, reviews, meta-analyses, case reports (or less than 10 participants in one of the surgical approach groups), or if no full text was available. Consensus on inclusion and exclusion was reached through discussion between the authors. If no consensus could be reached, a third author was consulted. References and citating articles were screened for additional studies.

### Quality assessment

The methodological quality of the studies was independently assessed by two authors using the risk of bias in non-randomized studies—of interventions (ROBINS-I) tool, to assess the risk of bias in the included studies [24]. Consensus on quality assessment was reached after discussion between the authors.

### Data extraction

Study characteristics and outcome data of the included studies were extracted. Additionally, the following data were extracted: type of surgical approach, type of surgical procedure when undergoing RS tumor resection, number of patients who underwent surgery for CPA tumor, sex, mean age, tumor size, and duration of follow-up.

### Statistical analysis

For calculation of *p*-values, the chi-square test was used.

## Results

### Search results and selection process

**Fig. 1:**
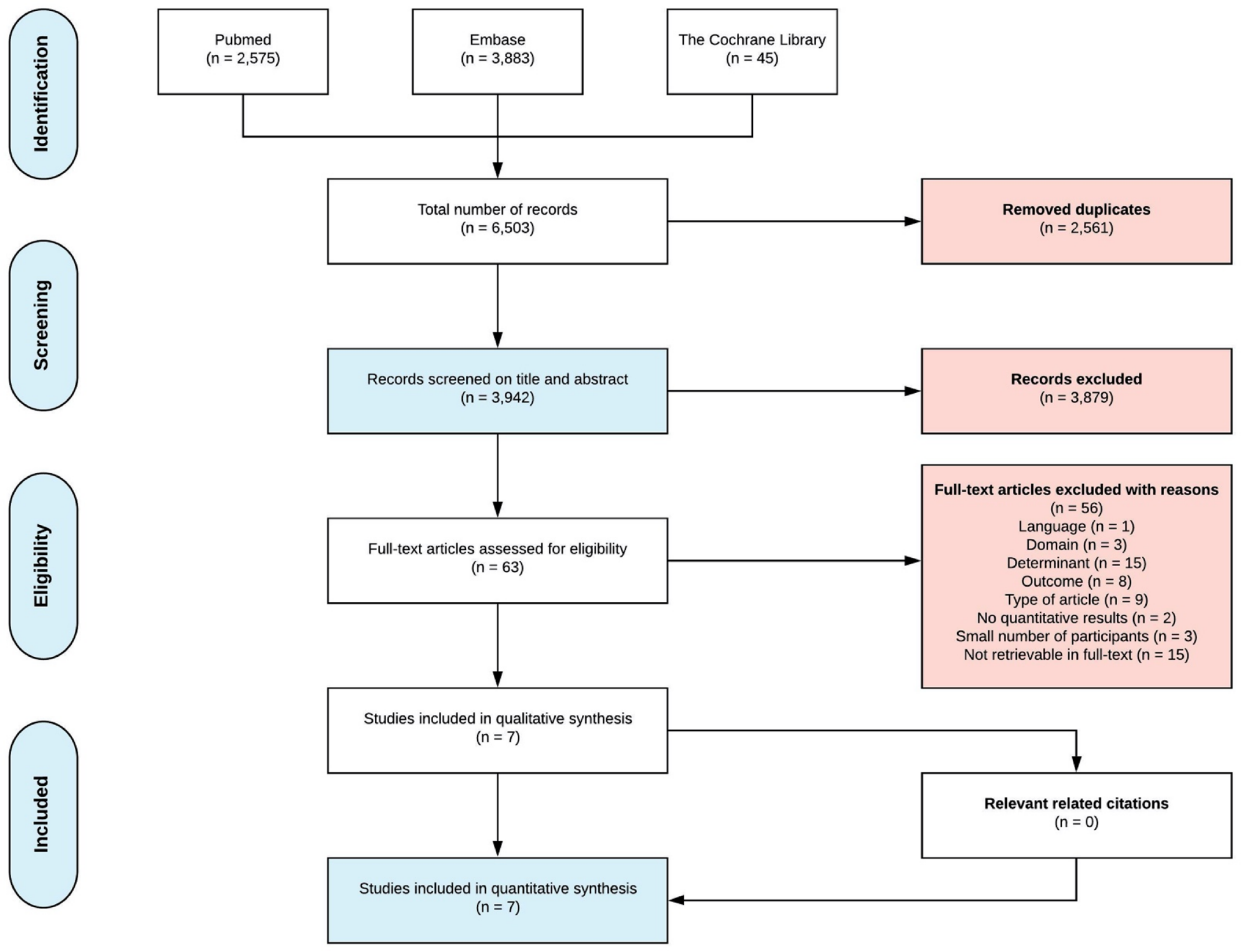
Flowchart of inclusion of relevant publications

A flowchart with the performed selection process is shown in Fig. 1. We retrieved a total of 3,942 articles after removing duplicates. After title and abstract screening, 63 articles were assessed for eligibility in full text. In total, seven studies were deemed eligible and critically appraised: six retrospective cohort studies and one cross-sectional observational study [25–31]. Reviewing of references and citation tracking did not result in additional relevant articles.

### Quality assessment

The results of the critical appraisal are shown in Table 1. Overall, one study scored a moderate risk of bias, and six studies scored a serious risk of bias.

**Table 1:** Critical appraisal

Article	Confounding	Selection of participants	Classification of interventions	Deviations from intended interventions	Missing data	Measurement of outcomes	Selection of the reported results	Overall risk of bias
Carlson 2015	L	S	L	L	NI	M	L	Serious
Levo 2000	L	S	L	L	L	M	L	Serious
Pedrosa 1994	L	S	L	L	NI	M	L	Serious
Rameh 2010	L	S	L	L	NI	M	L	Serious
Ruckenstein 1996	L	L	L	L	NI	M	L	Moderate
Ryzenmann 2004	L	S	L	L	NI	M	L	Serious
Schessel 1992	L	S	L	L	NI	M	L	Serious

*L *low risk of bias; *M* moderate risk of bias; *S* serious risk of bias; *NI* no information

### Baseline characteristics of included studies

The baseline characteristics of the included studies are shown in Table 2. All studies are similar regarding in- and exclusion criteria with the exception of Ryzenman et al. and Schessel et al., who included children and did not quantify the exact number of patients in this group [27, 31].

**Table 2:** Baseline characteristics of the included studies

Study	Design	Outcome measure	Outcome assessment	Treatment	Participants (N)	Surgical closure technique	Sex (m/f)	Mean age (range)	Tumor size (cm^3^/ stage, N)	Follow-up years ± SD (range)
Carlson 2015	Cross-sectional observational study	HDI, VS-symptom questionnaire	Postal/ hardcopy	TL	34	*NI*	*NI*	*NI*	*NI*	*NI*
				RS	94	*NI*	*NI*	*NI*	*NI*	*NI*
				Total	538^*a*^	*NA*	235/303	63.9 (55–72)	*NI*	7.7 (5.6–9.3)
Levo 2000	Retrospective cohort study	MPQ, Finnish Pain Questionnaire	Mailed	TL	23	*NI*	7/16	52.9	1.82	8.7
				RS	228	*NI*	95/133	47.1	2.15	8.6
				Total	251	*NA*	102/149	*NI*	*NI*	*NI*
Pedrosa 1994	Retrospective cohort study	Non-standardized disease-specific questionnaire	By telephone	TL	15	Fat graft	*NI*	*NI*	*NI*	*NI*
				RS	135	Craniectomy in 18 patients Cranioplasty^*b*^ in 117 patients	*NI*	*NI*	*NI*	*NI*
				Total	155^*c*^	*NA*	69/86	49 ± 12 (19–75)	*NI*	(8 m-10)
Rameh 2010	Retrospective cohort study	Non-standardized disease-specific questionnaire	Mailed	TL	58	*NI*	*NI*	*NI*	Stage III^*d*^, 26 Stage IV^*e*^, 33	*NI*
				RS	42	*NI*	*NI*	*NI*	Stage III, 36 Stage IV, 6	*NI*
				Total	101^*f*^	*NA*	44/57	55.19 ± 11.37 (21–90)	*NI*	5.9 ± 2.04 (2–9)
Ruckenstein 1996	Retrospective cohort study	Non-standardized disease-specific questionnaire	*NI*	TL	18	Fat graft	10/8	56	2.0	*NI*
				RS	35	Cranioplasty^*b*^	10/25	49	1.24	*NI*
				Total	53	*NA*	20/33	*NI*	*NI*	1 m, 6 m, 1y
Ryzenman 2004	Retrospective cohort study	Non-standardized disease-specific questionnaire	Mailed	TL	888	*NI*	*NI*	*NI*	*NI*	*NI*
				RS	493	*NI*	*NI*	*NI*	*NI*	*NI*
				Total	1651^* g*^	*NA*	569/1081^* h*^	58.2 (13–89)^*i*^	*NI*	*NI*
Schessel 1992	Retrospective cohort study	Non-standardized disease-specific questionnaire	By telephone	TL	40	Fat graft	21/19	45 (20–65)	1.5	5.0 (1–14)
				RS	58	Craniectomy	37/21	46 (16–64)	1.1	5.4 (8 m-14)
				Total	98	*NA*	58/40	*NI*	*NI*	*NI*

*N* number, *SD* standard deviation, *HDI* headache disability inventory, *VS* vestibular schwannoma, *TL* translabyrinthine, *RS* retrosigmoid, *NI* no information, *NA* not applicable, *MPQ* McGill pain questionnaire

^a^247 patients underwent stereotactic radiosurgery, 148 conservative observation, and 143 microsurgery whereof 15 patients received the middle fossa approach

^b^Cranioplasty with autologous bone graft

^c^For 5 patients the type of surgical approach could not be determined from the patient charts

^d^Defined as tumors 2–3 cm in diameter which contact brainstem and/or cerebellum

^e^Defined as tumors lager than 3 cm in diameter with compression on the brainstem and/or cerebellum]

^f^1 patient of the TL group was lost to follow-up

^g^96 patients underwent the middle fossa approach, and for 174 patients the type of surgical approach was unknown or not reported

^h^For 1 patient, sex was not identified

^i^For 5 patients age was not reported

In total, the results of 2,161 patients were included. The TL approach was performed in 1,076 patients whereas 1,085 patients underwent the RS approach. All patients suffered from unilateral CPA tumors and the majority of participants were female (61.4%). The patient mean age was 53 years (range 13 to 90). Three studies provided the exact proportion of patients who underwent craniectomy or cranioplasty when undergoing CPA tumor surgery through the RS approach [25, 30, 31]. Of the 228 participants undergoing the RS approach in these studies, 152 underwent cranioplasty with autologous bone graft and 76 craniectomy. Levo et al. used the RS approach for larger CPA tumors, whereas Ruckenstein et al. and Schessel et al. used the TL approach for larger tumors [26, 30, 31]. The duration of follow-up of the included studies varied from 1 month to 14 years [25–31].

### Postoperative headache rates after TL versus RS approach

The outcomes of the included studies are presented in Table 3. In total, 23.4% (494 out of 2,108) of the patients experienced POH, 36.2% (380 out of 1,050) of the RS group and 10.8% (114 out of 1058) of the TL group. Three studies, by Levo et al., Ryzenman et al. and Schessel et al., reported a significantly lower prevalence of chronic POH in the TL group (*p* = 0.019, *p* < 0.001 and *p* < 0.001, respectively) [26, 27, 31]. Ruckenstein et al. found a significantly lower POH rate at one and six months after surgery in patients undergoing the TL approach [30]. One year after surgery, this difference was no longer present. Two other studies, by Pedrosa et al. and Rameh et al., reported that the TL approach was associated with a lower incidence of POH compared to the RS approach, no significant difference between the two surgical approaches was found (*p* = 0.12 and *p* = 0.59, respectively) [25, 28]. Carlson et al. included 128 patients, of which 94 (73.4%) in the RS and 34 (26.6%) in the TL group, and reported a 1.5% higher POH rate in patients undergoing the TL approach (*p* = 0.87) [29]. In the study by Pedrosa et al. both retrosigmoid craniectomy and cranioplasty with autologous graft were performed [25]. In the craniectomy group, 15 out of 18 (83%) patients reported headache, in comparison to 83 of the 117 (71%) patients in the cranioplasty group (*p* = 0.27).

**Table 3:** Comparison of POH rates after translabyrinthine versus retrosigmoid approach

Article	Outcome measure	RS, total *N*	Outcome yes, *N* (%)	TL, total *N*	Outcome yes, *N* (%)	Difference (%TL-%RS)	*p*-value^a^
Carlson 2015	HDI > 14	94	29 (30.9)	34	11 (32.4)	1.5%	0.871
Levo 2000	Self-reported headache	228	97 (42.5)	23	4 (17.4)	− 33.5%	0.019
Pedrosa 1994	Self-reported headache	135	98 (72.6)	15	8 (53.3)	− 19.3%	0.120
Rameh 2010	Self-reported headache	42	24 (57.1)	58	30 (51.7)	− 5.4%	0.592
Ruckenstein 1996	Self-reported headache	35	*NI*^*b*^	18	*NI*^*b*^	*NI*^*b*^	0.03^*c*^; 0.006^*d*^; 0.6^*e*^
Ryzenmann 2004	Self-reported headache	493	95 (19.3)	888	61 (6.9)	− 12.4%	< 0.001
Schessel 1992	Self-reported headache	58	37 (63.7)	40	0 (0.0)	− 63.7%	< 0.001

*POH *postoperative headache, *RS *retrosigmoid, *N *number,* TL *translabyrinthine, *HDI* headache disability inventory, *NI* no information

^a^Calculated using chi-square test

^b^No exact numbers given in the article

^c^*p*-value retrieved from the article, after 1 month follow-up

^d^*p*-value retrieved from the article, after 6 months follow-up

^e^*p*-value retrieved from the article, after 1 year follow-up

## Discussion

### Principal findings

The objective of our systematic review was to investigate differences in POH rates between translabyrinthine (TL) and retrosigmoid (RS) approaches for resection of cerebellopontine angle (CPA) tumors. Overall, 23.4% of the subjects had POH, 36.2% of the RS group compared to 10.8% of the TL group. The study by Ruckenstein et al. was not used for this calculation because of insufficient data. We found three studies in which no significant difference between the two approaches was found (*p* = 0.871, *p* = 0.120, *p* = 0.592) [25, 28, 29], and three studies in which a significantly lower rate of POH was found in the TL group compared to the RS group (*p* = 0.019, *p* < 0.001 and *p* < 0.001) [26, 27, 31]. One study showed a significantly lower POH rate in the TL group after one and six months, but not after one year [30].

Previously, two reviews investigated the frequency of postoperative complications following CPA tumor resection [9, 21]. Sabab et al. found six studies reporting a trend towards lower POH rates in patients treated with the TL approach, of which one study presented statistically significant evidence (*p* < 0.05) [9]. The review by Ansari et al., also found a significant difference in chronic POH rates in favor of the TL approach (*p* < 0.001) [21]. However, this result was based on only one study, and therefore can be biased by coincidence. Furthermore, in both reviews little information was given about the quality and contents of the used studies [9, 21]. Therefore, we solely included comparative studies investigating POH rates between both the RS and the TL approaches in our systematic review, and critically appraised the scientific quality of the studies. This way, the patient groups had a higher comparability between the two approaches.

Overall, the individual quality of the included articles was poor. Only one study scored a moderate risk of bias [30]. Accordingly, we considered this result to be more reliable, compared to the other six studies, which scored a serious overall risk of bias because no information was available on the exact follow-up time points in the two different surgical approaches [25–29, 31]. Therefore, those results need to be interpreted more carefully.

Another important factor that should be taken into consideration is sample size. Two of the studies that found significant differences between the approaches were the largest studies we included regarding total subject count [26, 27]. The relatively small sample sizes of the remaining studies could pose a bias that needs to be considered in the interpretation of the different study outcomes [25, 28–31]. Also, a difference should be noted between the outcome parameters. The study by Carlson et al. used a headache disability inventory (HDI) score > 14 as outcome measure, while the other studies scored ‘headache in general’ as outcome measure [29]. The headache disability inventory (HDI) score does not directly reflect the occurrence of headache (but rather the impact of headache on quality of life) which makes the results of the study by Carlson et al. and the other studies hard to compare. Furthermore, the studies we have included and analyzed all reported different durations of follow-up (range 1 months to 14 years). This could influence the results as is seen in the study by Ruckenstein et al., where a different result is found at 1 months and 6 months follow-up compared to 1 year follow-up [30]. However, because we included only seven studies, and data on aforementioned factors is missing in some studies, it is not possible to take these factors into account for this review. Furthermore, the difference in outcome parameters and follow-up durations made the data unsuitable for a meta-analysis.

### Hypothetical mechanisms underlying POH after RS versus TL

We hypothesized that POH occurs more frequently in patients treated with the RS approach than in those treated with the TL approach. One reason is that drilling into the IAC during RS tumor resection increases the risk of bone dust entering the posterior fossa causing tissue reactions and, possibly, irritative arachnoiditis [2, 4, 30–34]. Additionally, surgical incisions to the suboccipital musculature (i.e. m. occipitalis and m. trapezius) might consequently lead to ingrowth of these anatomical structures into the exposed dura in the postoperative course [2, 4, 30–34]. This healing process can result in postoperative adherences, leading to POH as well. Considering that, the applied surgical details of the reported RS procedure could have an influence on the results regarding POH. Several studies have reported lower rates of POH in subjects undergoing cranioplasty, rather than craniectomy [9, 25]. More precisely, bone replacement instead of solely removing it may reduce the occurrence of tissue reactions and postoperative adherences between musculature and dura which results in lower POH rates. In the TL procedure there is no such dural exposure to ingrowing musculature, nor is intradural drilling applied during the procedure. These are two interesting differences in surgical details which might have an influence on the outcome. Therefore, future clinical prospective studies with precise follow-up will be necessary to further confirm our hypothesis. They should distinguish carefully between the performance of cranioplasty and craniectomy, as this might be the confounding factor that makes the RS approach with only craniectomy more prone to the occurrence of POH. Moreover, it would be interesting to directly compare the occurrence rate of POH between the RS approach with only cranioplasty and the TL approach.

### Clinical and surgical implications and future perspectives

Studies on this subject are limited, and consist frequently of small sample sizes or have a serious risk of bias. Thus, for clinical practice we cannot make recommendations based on this review.

However, the two largest studies in this review show a significantly lower rate of POH when using the TL approach, compared to the RS approach [26, 27]. The study by Ruckenstein et al. has a relatively small sample size, but a moderate risk of bias, and shows significantly lower rates of POH at two of the three measured time points in favor of the TL approach. Furthermore, the differences in POH rates in the aforementioned studies are relatively large. This, combined with the burden of chronic headache, make the possible difference in POH rates between the two approaches clinically relevant. We believe that this is enough reason for further prospective research, with proper methodology to ascertain if this is an existing phenomenon and parameter the surgical team should take into account. And if so, how large the difference in POH rates is. We performed a sample size calculation for a significance level of 95% and a power of 80% for different expected POH rates, based on our current results (Table 4).

**Table 4:** Sample size calculation

Ratio N of subjects RS:TL	2	1	0.5
Levo 43% POH in RS 17% POH in TL	128	112	120
Ryzenmann 19% POH in RS 7% POH in TL	324	276	297
Average^*a*^ 48% POH in RS 27% POH in TL	215	188	209
Total^*b*^ 36% POH in RS 11% POH in TL	119	102	109

Sample size calculation based on an α of 0.05 and a power of 80% (β = 0.2) using Fleiss’ method with continuity correction [35]. In the rows, the expected values of headache rates for both surgical methods are given, based on the results of the two largest studies included in this review, and on the average and total postoperative headache rates calculated using six of the seven included studies (Ruckenstein 1996 excluded because of insufficient information). In the columns the ratio of the number of subjects in the RS and in the TL group are given. The displayed sample sizes are the total numbers of participants needed, given the expected postoperative headache rates and the ratio of subjects in the RS and the TL groups

*N* number, *RS* retrosigmoid, *TL* translabyrinthine, *POH* postoperative headache\

^a^Average rates of POH calculated using six of the seven included studies (Ruckenstein 1996 excluded because of insufficient information)

^b^Rates of POH calculated using the total numbers of participants in six of the seven included studies (Ruckenstein 1996 excluded because of insufficient information)

## Strengths and limitations

The main strengths of our study are the systematic approach and our comprehensive search strategy, which allowed us to identify all relevant articles and available data from the literature. The main limitation is that we had to exclude 15 possibly relevant studies, because there were no full texts available even after consulting a scientific librarian. Also the retrospective character of the included studies and mostly unfixed postoperative follow-up periods are important setbacks.

## Conclusion and Recommendation

In conclusion, the results of this systematic review show some evidence of a lower rate of chronic postoperative headache when using the translabyrinthine approach over the retrosigmoid approach for benign CPA tumor surgery. Further prospective research is advocated to elucidate this important topic.

## Appendix

See Table 5.

**Table 5:** The performed search on Cochrane, PubMed and EMBASE (19–10-2020)

Database	Terms	Hits
Pubmed	(((Vestibular[Title/Abstract] OR Acoustic[Title/Abstract] OR Cerebellopontine[Title/Abstract] OR Cerebellopontine[Title/Abstract])) AND (Schwannoma*[Title/Abstract] OR Neuroma*[Title/Abstract] OR Neurinoma*[Title/Abstract] OR Neurilemoma*[Title/Abstract] OR meningioma*[Title/Abstract] OR tumor*[Title/Abstract])) OR (neuroma, acoustic[MeSH Terms] OR meningioma[MeSH Terms]) AND ((Retrosigmoid[Title/Abstract] OR RS[Title/Abstract] OR Translabyrinthine[Title/Abstract] OR TL[Title/Abstract] OR Suboccipital[Title/Abstract]) OR (headache [Title/Abstract]))	2,575
Embase	(((‘Vestibular’:ti,ab,kw OR ‘Acoustic’:ti,ab,kw OR ‘Cerebellopontine’:ti,ab,kw OR ‘Cerebello-pontine’:ti,ab,kw) AND (‘Schwannoma*’:ti,ab,kw OR ‘Neuroma*’:ti,ab,kw OR ‘Neurinoma*’:ti,ab,kw OR ‘Neurilemoma*’:ti,ab,kw OR ‘Meningioma*’:ti,ab,kw OR ‘Tumor*’:ti,ab,kw)) OR (‘acoustic neuroma’/exp OR ‘Meningioma’/exp OR ‘pons angle tumor’/exp)) AND ((‘Retrosigmoid’:ti,ab,kw OR ‘RS’:ti,ab,kw OR ‘Translabyrinthine’:ti,ab,kw OR ‘TL’:ti,ab,kw OR ‘Suboccipital’:ti,ab,kw) OR (‘headache’:ti,ab,kw))	3,883
Cochrane	((Vestibular OR Acoustic OR Cerebellopontine OR Cerebellopontine) AND (Schwannoma* OR Neuroma* OR Neurinoma* OR Neurilemoma* OR meningioma* OR tumor*)) OR Neuroma, acoustic OR Meningioma AND ((Retrosigmoid OR RS OR Translabyrinthine OR TL OR Suboccipital) OR (headache))	45
Total number of articles found = 6,503		
Number of articles after deduplication = 3,942		
